# Editorial: Brain stimulation in cognition and disease

**DOI:** 10.3389/fnins.2023.1213272

**Published:** 2023-05-16

**Authors:** Kai Yu, Jing Wang, Haiteng Jiang, Pierre D. Mourad

**Affiliations:** ^1^Department of Biomedical Engineering, Carnegie Mellon University, Pittsburgh, PA, United States; ^2^Department of Neurology, University of Minnesota, Minneapolis, MN, United States; ^3^Department of Neurobiology, Affiliated Mental Health Center & Hangzhou Seventh People's Hospital, Zhejiang University School of Medicine, Hangzhou, China; ^4^Division of Engineering and Mathematics, University of Washington, Bothell, WA, United States; ^5^Department of Neurological Surgery, University of Washington, Seattle, WA, United States

**Keywords:** brain stimulation, cognition, neurological disease, deep brain stimulation, transcranial direct current stimulation, transcranial alternating current stimulation, transcranial magnetic stimulation, transcranial ultrasound stimulation

## Introduction

Brain stimulation techniques have emerged as a promising avenue for both understanding the intricacies of cognitive processes and addressing neurological disorders. Our Research Topic of *Brain stimulation in cognition and disease* hosted by *Frontiers in Neuroscience* therefore focuses on the advancements, innovations, challenges, and future directions of brain stimulation technologies in cognition and disease.

Thanks to the effort of all the authors and invited reviewers, we have collected 11 articles with seven original research and four review papers. Their topics range from invasive to non-invasive brain stimulation modalities, from preclinical studies to clinical practices, from mechanistic insights of brain stimulation to treating brain diseases. Here, we provide commentary that highlights and offers a view toward the future of the exciting work offered by our Research Topic.

## Brain stimulation for improving cognition

Non-invasive brain stimulation (NIBS) shows great potential for improving cognition, by targeting specific cognitive functions guided by electrophysiological biomarkers.

In a rodent study, Xie et al. proposed a novel closed-loop transcranial ultrasound stimulation (TUS) protocol for targeted neuromodulation in the CA1 region of hippocampus. They reported differential effects from TUS triggered at the peak vs. the trough of the CA1 theta oscillatory activity, observing changes in many measures of theta- and gamma-band activity, including coupling between the bands. Theta rhythm has been associated with attention, information processing, decision making, memory consolidation, etc., while gamma activity can relate to execution of motor and memory tasks. Therefore, their closed-loop TUS protocol may 1-day improve specific memory and cognitive functions in humans.

In a human study, Guo et al. demonstrated that multitarget high-definition transcranial direct current stimulation (HD-tDCS) applied over the right inferior frontal gyrus and pre-supplementary motor area improved response inhibition and neural efficiency compared to single target HD-tDCS. Guo et al. also showed that repeated multitarget HD-tDCS plus cognitive training further improved response inhibition, especially in the high-performance subject group. Future studies should obtain fine-grained segmentation of the interested brain regions in order to develop a personalized multitarget stimulation protocol.

Wang et al. reviewed the current state of research on NIBS techniques for the treatment of stroke survivors. A key finding of this review is that repetitive transcranial magnetic stimulation (rTMS) and tDCS each offer significant promise for improving cognitive function. However, the authors also emphasize the substantial heterogeneity in the included studies regarding stimulation parameters, outcome measures, and patient characteristics, which limits the generalizability of the findings, important topics for improving future research in this area.

Moretti et al. offer a cautionary tale regarding two promising NIBS technologies: rTMS and transcranial alternating current stimulation (tACS) as applied over right posterior parietal cortex. They observed no significant effect from either stimulation modality on temporal and visuospatial attention. Despite the null findings, the authors' work refines our knowledge of the boundaries of these NIBS techniques by emphasizing the importance of optimizing the targeting and NIBS parameters to obtain effective neuromodulation.

A little studied but very exciting application of NIBS is optimization of inter-brain neuromodulation for improving teamwork. Lu et al. performed a review of this topic, emphasizing the neural mechanisms of teamwork and potential transcranial electrical stimulation (TES) related technologies to improve teamwork. While much of the available literature focuses on military pilots, improved teamwork has important applications beyond this cohort. The authors discussed the characteristics and existing usage of TES. They found that inter-brain synchronization (IBS) might underlie consistent behaviors or intentions between persons, hence use of TES to enhance IBS might promote cooperation. To further increase IBS, the authors proposed using hyper-tACS together with hyper-scanning technology to enhance teamwork.

## Brain stimulation for treating brain disease

The usage of brain stimulation for treating neurological and psychiatric diseases has also grown significantly and researchers continue to seek improvements in existing techniques and development of novel stimulation approaches for better clinical outcome.

Lin et al. demonstrated a rescue procedure involving bilateral subthalamic nucleus (STN) deep brain stimulation (DBS) together with posteroventral pallidotomy (PVP) for dystonia patients experiencing secondary failure of DBS in the globus pallidus internus (GPi). All six participants in the study experienced reduced motor benefits from bilateral GPi DBS 12–24 months after standard DBS. Their approach provided significant improvement in both the movement and disability scores with PVP + bilateral STN DBS that lasted for at least 12–24 months. Further study with more patients may 1-day show that their protocol can offer an important treatment option for these patients.

Wu et al. explored the potential synergistic benefit of combining high-frequency repetitive transcranial magnetic stimulation (HF-rTMS) and cervical nerve root magnetic stimulation (CNRMS) to improve motor function in the upper extremities of stroke patients. The observed promising immediate post-intervention effects motivate future research that examines the long-term effect of their novel approach. This preliminary study holds promise for enhancing rehabilitation strategies for stroke survivors and offers valuable insights into the mechanisms underlying motor recovery.

Xu et al. investigated the effect of rTMS on serum levels of serum amyloid A (SAA) and testosterone in a real-world setting. The authors found that patients with depression benefit most from combined rTMS treatment with medications. Future research is needed in the form of double-blind, randomized control trials that examines the relationship between SAA level and rTMS depression outcome.

Ma et al. provided a systematic review and meta-analysis of the effect of tDCS for patients with disorders of consciousness, showing a significant increase in GCS (Glasgow coma scale) scores and CRS-R (Coma Recovery Scale—Revised) scores due to repeated application of tDCS, especially for patients in a minimal conscious state (MCS). While supportive, the authors could not identify optimal stimulation parameters due to the limited number of eligible studies and wide range of stimulation protocols.

He et al. provided a mechanistic overview of tACS, *in vivo*, as a means of optimizing its parameters to improve its efficacy and broaden its applications. The authors argued that future directions for tACS need to take into account, in a systematic fashion, the frequency, spatial, mechanism-specificity of tACS as well as robustness and replicability of associated findings.

## Conclusion

The future of brain stimulation in cognition and disease is rich with possibilities. Multidisciplinary collaborations between neuroscientists, engineers, and clinicians can drive innovation and accelerate the translation of research findings into practical applications. The dissemination of knowledge through open access journals, such as *Frontiers in Neuroscience*, is vital for fostering collaboration and ensuring that the benefits of brain stimulation research reach a wide audience. We believe the publications collected here will become an important resource for those interested in this research realm.

## Author contributions

All authors listed have made a substantial, direct, and intellectual contribution to the work and approved it for publication.

